# Childhood Kaposi's sarcoma: clinical features and therapy.

**DOI:** 10.1038/bjc.1976.88

**Published:** 1976-05

**Authors:** C. L. Olweny, A. Kaddumukasa, I. Atine, R. Owor, I. Magrath, J. L. Ziegler

## Abstract

**Images:**


					
Br. J. Cancer (1976) 33, 555

CHILDHOOD KAPOSI'S SARCOMA:

CLINICAL FEATURES AND THERAPY

C. L. M. OLWVENY, A. KADDUMUKASA, I. ATINE, R. ()WVOR,

I. MAGRATH* AND J. L. ZIEGLER*

Fromn the Uganda Cancer Institute an(t Department of Pathology,

Makerere University, Kampala, Uganda

Received 24 November 1975  Accepted 31 December 1975

Summary.-Twelve cases of childhood Kaposi's sarcoma seen at the Uganda Cancer
Institute over the last 7 years are reported. The disease presents mainly as
generalized lymphadenopathy, with sparsely and anomalously distributed cutaneous
nodules, and has a higher proportion of females with the disease than in the adult
form. The histology is usually of mixed cell type. If not treated, childhood Kaposi's
sarcoma runs a fulminating course, but disease control with chemotherapy is
associated with prolonged survival.

KAPOSI'S sarcoma is rare in childhood.
In a review of 1256 cases by Dutz and
Stout (1960), only 40 (3-2%) were under
6 years. Eighteen of the 40 cases (450o)
were from the African continent alone.
The first acceptable case of Kaposi's
sarcoma in a child was described in a
monograph by de Amics in 1882, and
cited in a more recent paper by Ronchese
(1958).  Features  which  differentiate
childhood disease from the adult form
include the clinical presentation with
lymphadenopathy, the sparsely and ano-
malously distributed cutaneous nodules,
the high proportion of females and a
fulminatingl course if untreated. In a
previous report from this Institute, the
varied clinical and pathological features
of Kaposi's sarcoma were classified (Taylor
et al., 1971a). The lymphadenopathic
form was associated with the childhood
cases, but this group constituted a very
small proportion of the series. In this
paper we report in detail 12 cases of
Kaposi's sarcoma in children under 15
years of age seen at the Uganda Cancer
Institute (UCI) over the last 7 years.

PATIENTS AND METHODS

Details of presentation, clinical course,
response to treatment and other associated
features were obtained from the case records.
Ten children were referred to the Lymphoma
Treatment Centre with a provisional diag-
nosis of malignant lymphoma and two with
a histological diagnosis of Kaposi's sarcoma.
The histology sections were reviewed by
one of us (R.O.) and classified according to
the criteria described previously (Taylor et
al., 1971a).

We define complete remission as disap-
pearance of clinically measurable tumour,
partial response as a reduction in tumour
volume of at least 5000 and no response as
a lesser response, or disease progression.

RESULTS

The clinical details of the 12 patients
are given in Table I. The median age
at presentation is 8 years (range 11-15
years). There were 7 males and 5
females, giving a male: female ratio of
1X4: 1. Ten of the 12 patients presented
with lymphadenopathic disease; one had
a florid lesion and one had cutaneous
nodules. One patient (No. 3) had nodular
lesions apart from peripheral lymph-

* Curreint address: National Cancer Institute, National Institutes of Health, Bethesda, AMaryland
20014.

C. L. M. OLWENY E1 AL.

* C     *. (1    .     H          PL

6
S

-j-

I

elt-

-4-

00
S

I 0

I 0

6

S.

o

I    0

E

?

0I

0 -

0~~~~~~~~~~~~C ll

A-+ +
00      0   0

0-     0+   ol

I                    I               C;

6

I           V -    I

C) H

.1 11 11 11 11 11 11 11

+                ?            ~                        -

*              6         6                                     ?

I               E                           . E  C- E- E-- > - C-gS

o               cs Cc    eS      -

Fz        0 Z Fz0 ;     0   0

0
+-

0

C)

C.

V~

C.)

41    *

0   0  0 :

; C.)  C)

fi   Fi  Fi   ~~~~~C)

~~ = = = ,; .sZ .? ~~~d                             Cd

4               0                     0                   E

0                                           0~~~~~~~~~~~~4;

c           e  0                  Cs

SP -

T-      -_N     Fo~  X5  -,  m.nl-i  p

4          lewi  Ot  r-  *6  a

0        .      .      -

-        q      eS c:   - s

* E zL, = t  E;  Z ?   a W x

0 d        Ca

Ca~

CaP    ~ ~ i

e s c   Ii I   I Il I   i i

0   -

.                       .

O -

z +

*-7 C

HF,

?L          EH-

6
S

H      H     H   H
4           -4 +~  4

0     0       0 0 ?

V~~~~~~~~~~! V  VV

m

?% -C,' x ;-?

;'. C)

C) 0?   x.  ?=      m
tL          1-0

C?
m

C?          ?g
7-)

(:Ii

556

v-~

.

9 9I

CHILDHOOD KAPOSI S SARCOMA

(a)                                           (b)

FIG. l(a). Patient Z (No. 10) with bilateral mandibular swelling. (b).-Same patient Z (No. 10).

Close-up of mouth showing right lower quadrant gingival mass. He also had left upper gingival
mass not seen here.

adenopathy. One (No. 9) had visceral
disease, while another (No. 10) had
mandibular involvement (Fig. la and b).
The lymphadenopathy was often general-
ized but particularly marked in the
cervical region (Fig. 2) and the groin
(Fig. 3). Two had mediastinal widening
as shown by the chest roentgenograph
(Fig. 4). All 12 children had a mixed
cellular histological pattern.

The therapeutic approach varied, as
the 12 children were admitted when
different treatment protocols were under
study (Olweny et al., 1974; Vogel et al.,
1971).  The primary and subsequent
treatments given are detailed in Table I.
Six received actinomycin-D alone as their
primary treatment regimen, two received
a combination of actinomycin-D and

vincristine, three received a 3-drug com-
bination consisting of actinomycin-D,
vincristine and imidazole carboxamide
(DTIC), and one was treated originally
with cyclophosphamide. Doses and sche-
dules of chemotherapy are described in
detail in previous reports from this centre
(Olweny et al., 1974; Vogel et al., 1971).
Of the 6 patients treated with actino-
mycin-D alone, 2 (Nos. 8 and 10) attained
complete remissions and remained in
remission 50+ and 66+ months, 2 had
partial responses and 2 did not respond.
Of the 2 patients treated with actino-
mycin-D and vincristine combination,
one (No. 11) achieved complete tumour
regression but relapsed after 24 months
and the other (No. 4) did not respond.
The 3 patients (Nos. 1, 5 and 12), who

557

C. L. M. OLWENY ET AL.

FIG. 2.-Patient M. (No. 4), showing marked

cervical lymphadenopathy. Scars are from
therapy prior to hospitalization.

were treated with the actinomycin-D,
vincristine and imidazole carboxamide
combination all achieved complete tu-
mour regression with remissions lasting
3, 6, and 12 months respectively. The
single patient who received cyclophos-
phamide alone did not respond.

Treatment of relapse included: radio-
therapy (3); BCNU (2); actinomycin-D
and vincristine (3); actinomycin, vin-
cristine and DTIC (4); and DTIC and
bleomycin (1). As can be seen in Table
I, the most effective combination in
inducing a remission appears to be
actinomycin-D, vincristine and DTIC.
All 4 patients who later received this
combination, having either relapsed or
responded incompletely to other forms
of treatment, responded with complete
tumour regression lasting 6+, 9, 12 and
13 months. In the majority of patients
relapse was at the same site as the
original tumour suggesting incomplete
eradication of tumour.

FIG. 3.-Patient W. (No. 7), with prominent

bilateral groin lymphadenopathy.

FIG 4.-Chest roentgenograph of patient A.H.

(No. 1) showing the widening of the media-
stinum.

558

CHILDHOOD KAPOSI S SARCOMA

Of the 12 patients studied, 2 have
died, 3 are lost to follow up, 3 are alive
and free of disease, and 4 are alive with
active disease. The 2 deaths occurred at
1 month and 51 months respectively.
Excluding the 3 patients lost to follow-up,
the median survival is 46 months (range
1-68).

DISCUSSION

Kaposi's sarcoma though rare in most
parts of the world is relatively common
in tropical Africa. Childhood cases are
particularly uncommon and the majority
reported are from the Continent of Africa
(Dutz and Stout, 1960). The disease in
children has certain atypical features.
First, the mode of presentation with
generalized lymphadenopathy is almost
always confined to childhood cases. Ten
out of 12 children in this series presented
with moderate to marked lymphadeno-
pathy. The presentation with lymph
node enlargement appears to be a feature
commoner in Negro children than in
Caucasians. In the 40 cases reviewed by
Dutz and Stout, 8 out of 18 (44.4%)
African children had lymphadenopathy
only while only one of the 22 (4-5%)
non-Africans presented with similar fea-
tures (Dutz and Stout, 1960). In a
more recent report of 51 autopsies in
childhood cases from East Africa, 20 had
lymph node enlargement, 5 had skin
nodules as well as lymph node enlarge-
ment, and 2 had lymph node and ocular
involvement as the major clinical features
of the disease (Slavin et al., 1970). The
skin nodules when present are sparse and
distributed over anomalous sites. The
eyelids, lacrimal glands, jaw, parotid and
salivary glands are areas of predilection
and sometimes patients present with
Mickulicz syndrome. The reason for pro-
pensity to glandular involvement in
African children is not clearly understood.
Some authors have tended to regard
Kaposi's sarcoma as a disease arising
in the reticuloendothelial system. This
is based on the observation of occasional
co-existence of Kaposi's sarcoma and

lymphoproliferative disorders such as
Hodgkin's disease, lymphosarcoma, my-
cosis fungoides and leukaemia (Cox and
Helwig, 1959; Moertel, 1966). However,
this association is more commonly seen
in adult whites than in Africans, while
lymph node disease in general is com-
moner in the Africans than in whites.
The presentation with generalized lymph-
adenopathy may lead to wrong diagnosis.
Tuberculosis and/or Hodgkin's disease
are the frequent referring diagnoses; both
being prevalent in tropical Africa. Ten
of the patients reported here were referred
with a provisional diagnosis of malignant
lymphoma.    Kaposi's sarcoma should
therefore be included in the differential
diagnosis of any child with lymph node
enlargement.

A second difference between childhood
and adult Kaposi's sarcoma is the greater
frequency of females with the disease.
Most authors report a male: female ratio
of 3: 1 (Dutz and Stout, 1960; Slavin et
al., 1970; Templeton, 1972). This is
in marked contrast to the adult series
where the ratio is 13: 1 (Olweny et al.,
1974). In the present series the male:
female ratio is almost 1 : 1. So far there
is no satisfactory explanation for the
sex distribution. Sex hormones have
been suggested as possibly protecting the
post-pubertal females, but treatment with
oestrogens has yielded no beneficial results,
and some patients have developed the
disease while pregnant (Taylor et al.,
1971b). However, review of childhood
cases in Uganda prior to 1967 revealed
that all 9 recorded cases were males
(Davies and Lothe, 1962).

The histological features observed in
this small series also deserve mention.
All 12 patients had the mixed cell type
histological picture which is consistent
with an earlier report where lymph-
adenopathic disease was also exclusively
of mixed cellular type (Taylor et al.,
1971b). This histological appearance oc-
curs overall in some 70% of adult Kaposi
sarcoma patients in Uganda (Olweny et
al., 1974).

559

560                     C. L. M. OLWENY ET AL.

It is generally agreed that lymph-
adenopathic childhood Kaposi's sarcoma
is disseminated and aggressively malig-
nant. If not treated, death will almost
certainly ensue within one year. The
response to treatment has been varied
and difficult to assess as no uniform
approach has been used. However, two
patients treated only with actinomycin-D
are alive and apparently disease free at
52 +  and 68 +  months respectively, so
that there is no doubt that chemotherapy
alone can be highly effective therapy.
The combination of actinomycin-D, vin-
cristine and DTIC may prove to be even
more effective since it has induced com-
plete remission in 3 out of 3 patients
when used as primary therapy and 4 out
of 4 patients with recurrent tumour.
In one of these patients this combination
was used after a partial response had
been induced by radiotherapy, and the
latter form of treatinent, although used
only for treatment of tumours not re-
sponding or responding partially to chemo-
therapy, has also proved useful in
the few patients who received it. In
appropriate clinical circumstances radio-
therapy may further enhance the efficacy
of chemotherapy, and until further data
become available, a combination of radio-
therapy (where facilities exist) and the
three drug combination is probably the
treatment of choice.

The Uganda Cancer Institute is sup-
ported by a contract NO1-CM-71343 with
the National (Cancer Institute, National
Institute of Health, Bethesda, Md., USA.
Dr Magrath is recipient of a grant from
the Cancer Research Campaign of England.

We are grateful to Mr Sentainu
Majweega, to Mr P. Mbalire and Mr L.
Majweega for their assistance in the
follow-up of these patients. We also
wish to thank the nursing staff of LTC
who looked after the patients. Finally
we wish to thank Mr A. Wamala for the
secretarial assistance.

REFERENCES

Cox, F. H. & HELW1CG, E. B. ( 1959) Kaposi's

Sarcoma. Cancer, -N'.Y., 12, 289.

DAVIES, J. N. P. & LOTHE, F. (1 962) Kaposi's

Sarcoma in AfIrican Children. Actdt I T7ino Ititerniat.
Contra (ancrum, 18, 394.

DUTZ, W. & STOITT, A. P. (1 960) Kaposi's Sarcoma

in Infants arndi Children. Catncer, N. Y., 13,
684.

MOERTEL, C. G. (1'966) 'Multiple Primary Malignant

Neoplasms. In   Recen-t Adc-ances ini Canticer
Reseairch, Vol. 7. Berlin: Springer.

OLWENY, C. M., ToYA, T., KATON( OLE-AMBIDDE,

E., LWANGA, S. K., OWOR, R., KYALWAZI, S. K.
& VOGEL, C. L. (1974) Treatment of Kaposi's
Sarcoma   by  Combination- of Actinomycin-D
Vincristine ain(d Imidlazole CaIboxami(le (NSC-
45388); Results of a Randomized Clinlical Trial.
IJt. J. Caricer, 14, 666.

RONCHESE, F. (1958) Kaposi's Sarcoma; Overlooked

Essay of 1882. Archs Derm., 77, .542.

SLAVIN, G., CAMERON, H. MIc'D., FORBES, C. &

MITCHELL, R. M. (1970) Kaposi's SaIrcoma in
East African ChildreIn: 51 Cases. J. Path.,
100, 187.

TAYLOR, J. F., TEMPLETON, A. C., V'OG'EL, C. L.,

ZIEGLER, J. L. &   KYALWAZI, S. K. (1971a)
Kaposi's Sarcoma in UgaInda: A Clinico-patho-
logical Studly.  mit. J. Cancer, 8, 122.

TAYLOR, J. F., TEMPLETON, A. C., KYALWAZI, S. K.

&  LITBECA, A. (1971b) Kaposi's Sarcoma in
Pregnancy. Two Case Reports. Br. J. Surg.,
58, 577.

TEMPLETON, A. C. (1972) Stucdies ill Kaposi's Sar-

coma; Postmortem Findings anid( Disease Patter-n
in Women. Cantcer, N. Y., 30, 854.

VOG1EL, C. L., TEMIPLETON, C. J., TEMPLETON, A. C.,

TAYLOR, J. F. & KYALWAZI, S. K. (1971) Treat-
ment of Kaposi's Sarcoma with Actinomycin-D
andt Cyclophosphami(le: Results of a Rand(lomizedl
Cliinical Trial. Im2t. *I. C'anicer. 8, 136.

				


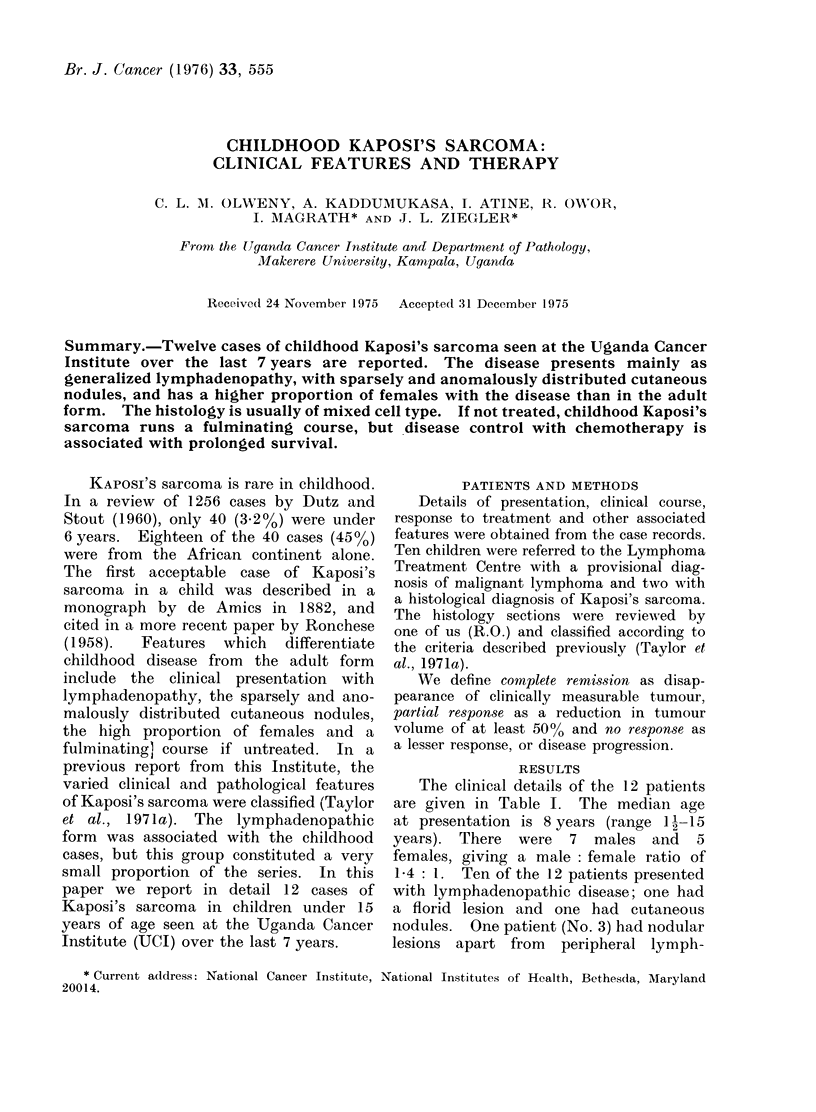

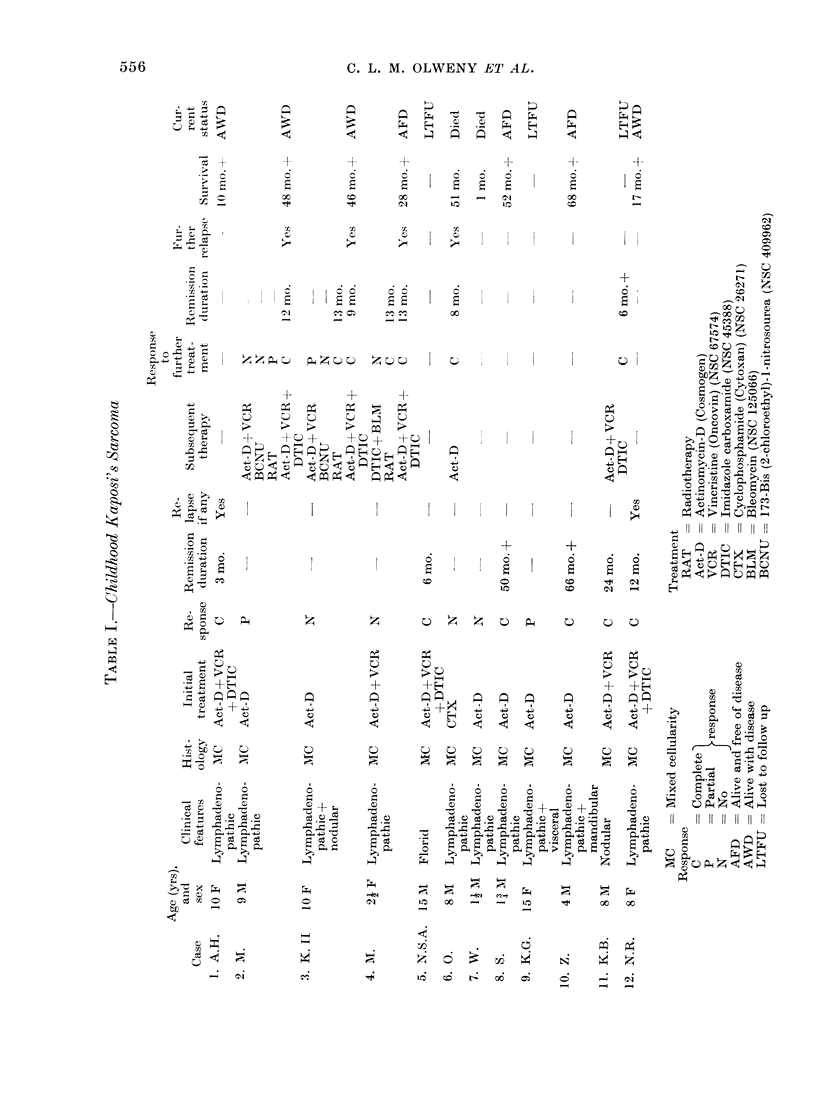

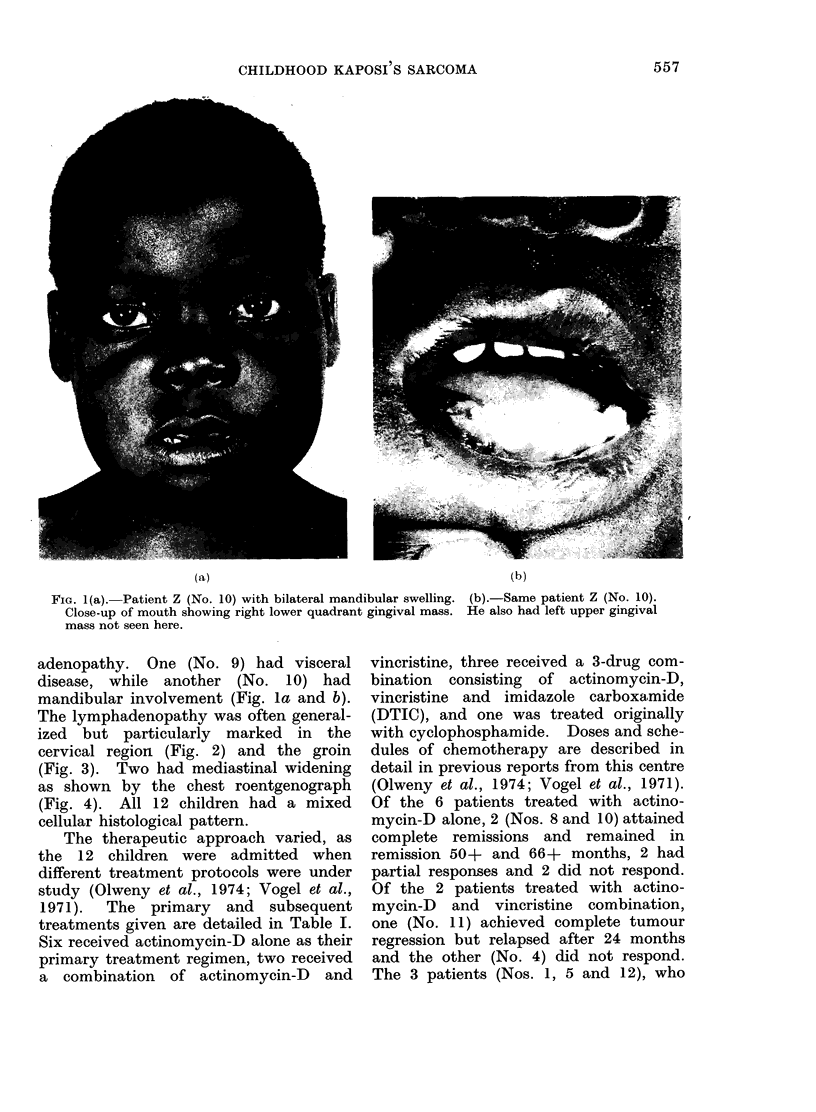

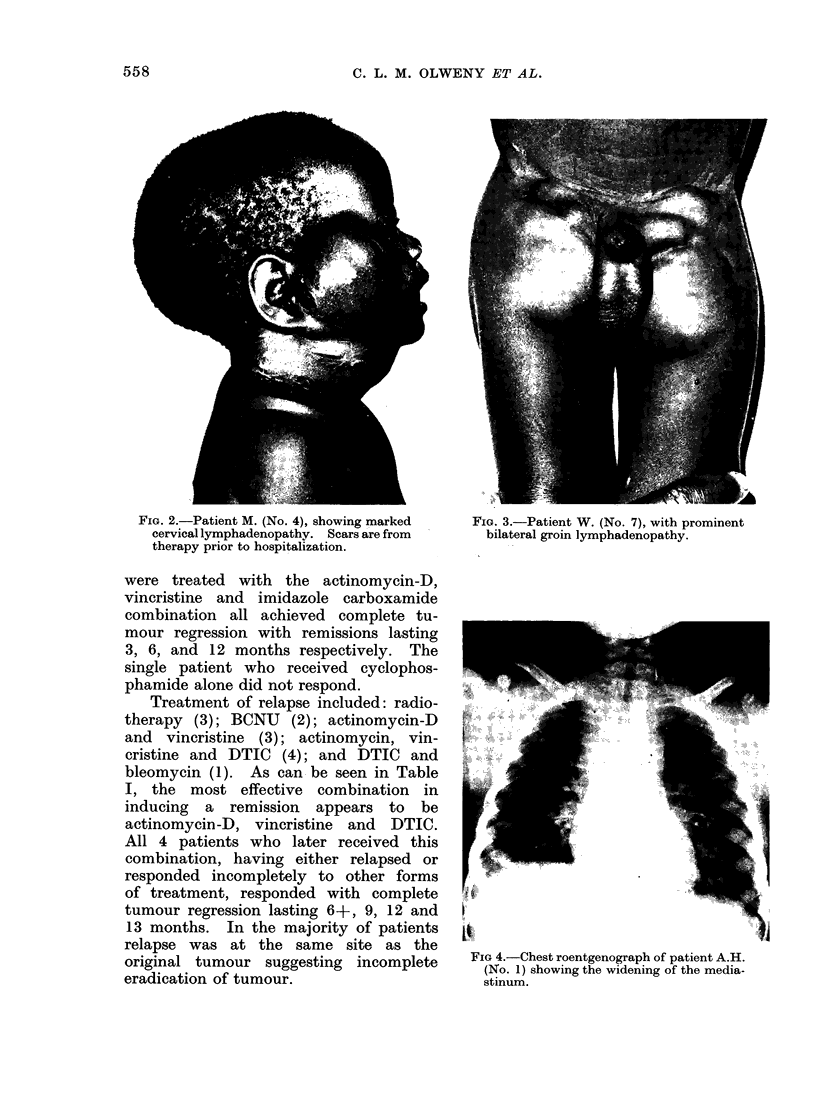

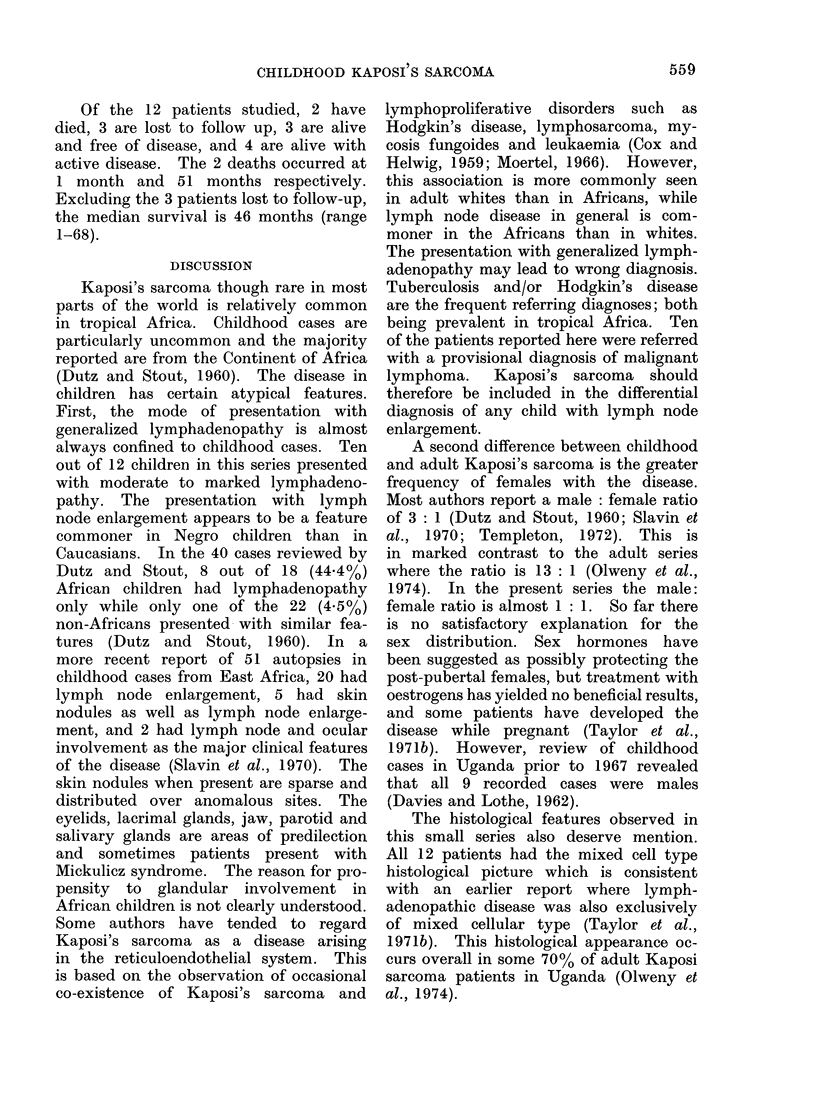

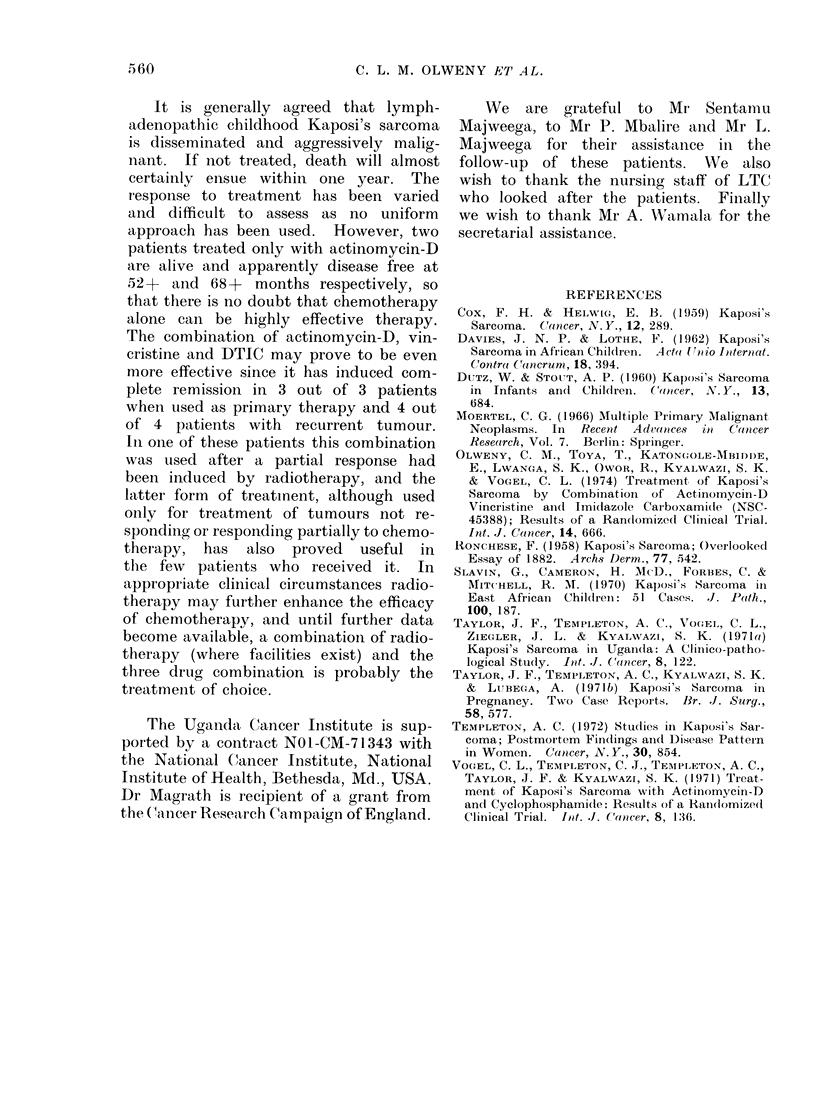

